# Beyond classification: gene-family phylogenies from shotgun metagenomic reads enable accurate community analysis

**DOI:** 10.1186/1471-2164-14-419

**Published:** 2013-06-22

**Authors:** Samantha J Riesenfeld, Katherine S Pollard

**Affiliations:** 1 Gladstone Institute of Cardiovascular Disease, University of California, San Francisco, CA, 94158, USA; 2 Division of Biostatistics and Institute for Human Genetics, University of California, San Francisco, CA, 94158, USA

**Keywords:** Phylogenetics, Metagenomics, Simulations

## Abstract

**Background:**

Sequence-based phylogenetic trees are a well-established tool for characterizing diversity of both macroorganisms and microorganisms. Phylogenetic methods have recently been applied to shotgun metagenomic data from microbial communities, particularly with the aim of classifying reads. But the accuracy of gene-family phylogenies that characterize evolutionary relationships among short, non-overlapping sequencing reads has not been thoroughly evaluated.

**Results:**

To quantify errors in metagenomic read trees, we developed MetaPASSAGE, a software pipeline to generate in silico bacterial communities, simulate a sample of shotgun reads from a gene family represented in the community, orient or translate reads, and produce a profile-based alignment of the reads from which a gene-family phylogenetic tree can be built. We applied MetaPASSAGE to a variety of RNA and protein-coding gene families, built trees using a range of different phylogenetic methods, and compared the resulting trees using topological and branch-length error metrics. We identified read length as one of the major sources of error. Because phylogenetic methods use a reference database of full-length sequences from the gene family to guide construction of alignments and trees, we found that error can also be substantially reduced through increasing the size and diversity of the reference database. Finally, UniFrac analysis, which compares metagenomic samples based on a summary statistic computed over all branches in a read tree, is very robust to the level of error we observe.

**Conclusions:**

Bacterial community diversity can be quantified using phylogenetic approaches applied to shotgun metagenomic data. As sequencing reads get longer and more genomes across the bacterial tree of life are sequenced, the accuracy of this approach will continue to improve, opening the door to more applications.

## Background

Phylogenetic trees are an important component of a wide variety of biological analyses, from genome annotation to community ecology. Much attention has been paid to the problem of estimating accurate phylogenies from DNA and amino acid sequence data, including the development of statistical methods to quantify support for each bifurcation in a tree. Until recently, phylogenetic methods have largely been applied to sequence data obtained through genome projects and targeted sequencing of individual genes using PCR. For these reasons, bacterial phylogenetics has been constrained to studies of specific genes or to cultured organisms, which represent a small and biased portion of the bacterial tree of life.

Shotgun metagenomics, the high-throughput sequencing of fragmented total DNA from a sample, gives an essentially random collection of DNA sequences from the genomes of the organisms present in a sample. These data provide an opportunity to analyze the full taxonomic and functional diversity encoded in the genomes of microbial communities. Metagenomic studies of natural environments, built environments, and the microbiomes of plants and animals have identified thousands of novel proteins and organisms. In order to annotate and interpret these discoveries, it is important to place them in the context of current knowledge using phylogenetic techniques. For example, UniFrac analysis of gene-family phylogenies identified convergent evolution of carbohydrate-active enzymes in human gut communities [1]. Another study showed that blooms of closely related species cause the phylogenetic diversity of marine microbes to be much lower in surface waters compared to below the photic zone at the HOT/ALOHA study site, despite the fact that communities at different depths have similar numbers of operational taxonomic units (OTUs) [2]. Similar insights have been gained by comparing protein abundance and phylogenetic diversity. For instance, relative abundance of common metabolic pathways is relatively stable across human microbiomes, whereas the phylogenetic diversity of the protein families encoded in these and less common pathways is more variable and possibly associated with host phenotypes [3]. Testing hypotheses about diversity requires a reliable technique for quantifying the phylogenetic relationships amongst shotgun metagenomic sequencing reads.

In contrast to the well-resolved approaches to analyze PCR-targeted small-subunit ribosomal RNA (rRNA) sequences [4], new techniques are needed for phylogenetic analysis of more complex shotgun metagenomic data. One goal of such methods is to leverage what is already known about gene families in order to exploit the information in short, non-overlapping read fragments, while taking into account the bias and potential error they introduce. Shotgun sequencing reads can often be identified as homologues of a gene family if there are a reasonable number of diverse sequences from the family in existing databases (e.g., [5]). Nonetheless, integrating reads into alignments and phylogenetic trees of full-length gene sequences, or generating alignments and trees for reads alone, remains a major bioinformatics challenge. If high quality trees could be built from shotgun metagenomic data, they would greatly expand our ability to characterize and compare microbial communities.

Phylogenetic methods typically depend either on the ability to assess evolutionary distance between sequences, e.g., based on computing percent identity, or to fit an evolutionary model to a sequence alignment [6]. The first approach is not possible using only the random pieces of each sequence present in a shotgun metagenomic sample. In the second case, likelihoods can be computed for models that include missing data, but the results have not been studied for situations in which missing data is as widespread as is observed in shotgun metagenomes. One solution is to analyze metagenomic reads via fragment recruitment [7], whereby a full-length gene sequence is identified as being the likely source of a shotgun-sequenced read or being closely related to the source, due, for example, to their BLAST-based similarity. The full-length sequence is then used in place of the read in downstream analysis. To work well, this approach requires a very complete reference database for the gene family, as well as reads that are long enough to be identifiable. Another approach to applying phylogenetic analysis to shotgun-sequenced data is to require the use of longer sequences assembled from reads [8]. But if the community sampled is complex and the read sequences are short, both common conditions, assembly is prone to producing chimeras [9,10]. Recent phylogenetic “placement” and “phylotyping” methods [11-14] place each read independently in a fixed phylogeny, with the aim of classifying reads with respect to a set of reference sequences. These methods have been tested for their accuracy in classification, rather than their effectiveness in creating phylogenetic trees of sequence reads.

**Figure 1:**
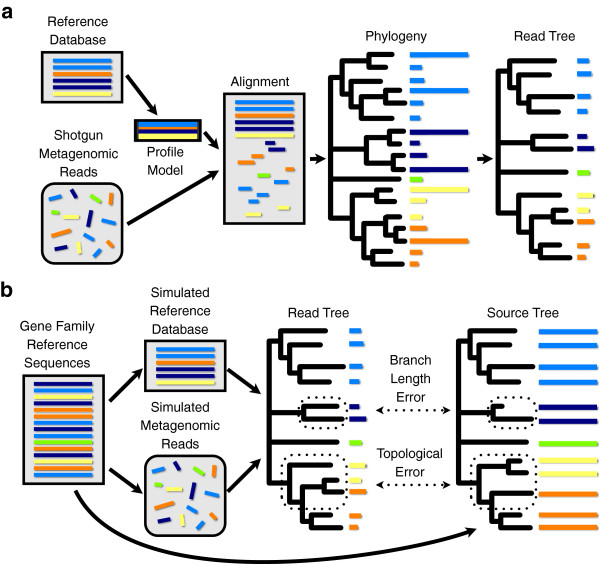
**Schematic for constructing metagenomic read trees and the simulations framework used to evaluate their accuracy. (a)** Construction of a phylogeny with leaves labeled solely by metagenomic reads. A reference database of full-length sequences is used to build a profile model. Metagenomic reads are aligned to the full-length reference sequences via the profile model. The alignment is used to build a phylogeny from which reference sequences are then pruned to create a read tree. **(b)** Schematic for the simulations framework used to evaluate the accuracy of read trees. For each gene family, we used MetaPASSAGE to sample two sets of full-length sequences: a simulated reference database and a sample of source sequences from which shotgun metagenomic reads were generated. We built read trees and then measured the accuracy of each read tree by comparing it to a source tree, labeled by the full-length gene sequences corresponding to the reads. Both branch-length errors and errors in topological relationships were assessed.

In this paper, we evaluate an approach that enables the direct phylogenetic analysis of shotgun metagenomic reads. The method uses a reference database of full-length sequences to guide construction of a phylogeny composed solely of the metagenomic reads from a gene family (“read tree”) (Figure 1). Phylogenetic triangulation enables non-overlapping reads to be related to each other via their relative distances from full-length sequences. The aim of this approach, and the basis on which we evaluate it, is to provide an estimate of the mutual relationships among all reads. While it has already proved successful for generating a phylogenetic distance measure to cluster 16S rRNA gene sequences into OTUs [15] and for quantifying the phylogenetic diversity of microbial communities [2] there are clearly limits to this approach. Extremely short reads and a low-coverage reference database are likely to result in very inaccurate trees. Most phylogenetic methods were not designed for and have not been evaluated on data sets that contain such an extent of missing data over random positions.

*In silico* simulations of metagenomic data provide a means to perform these evaluations in the absence of gold-standard data sets and fully characterized communities. Metagenomic simulation programs have been released [16,17], but none of these includes the automated processing of sequencing reads necessary for phylogenetic analysis. To address this problem, we extended the MetaSim software package [17] into a software workflow, called MetaPASSAGE, for streamlined creation and processing of metagenomic simulations. MetaPASSAGE enabled us to perform rigorous statistical assessments of metagenomic phylogenies (Figure 1). We quantified the effects of reference database composition, mean read length, number of reads, and phylogenetic algorithm on the accuracy of metagenomic read trees for 16S rRNA and four proteins. To contextualize our results, we included a limited investigation of the effect of sequencing error. We also explored the accuracy of statistical tests based on read trees through a simulated analysis using Fast UniFrac [18], which measures distance between communities using phylogenetic information. The results of our study show that reasonably accurate read trees can be constructed in many situations, although the magnitude of topological and branch-length error varies widely as a function of the parameters we explored. Nonetheless, it appears that statistical tests that use cumulative information from the whole read tree have low false positive rates and similar power to tests performed on phylogenies of full-length sequences, even when the read tree contains many errors.

## Results and discussion

### MetaPASSAGE: software for simulation and alignment of shotgun metagenomic reads

We developed MetaPASSAGE (https://github.com/sriesenfeld/MetaPASSAGE), i.e., Metagenomic Pipeline for Automated SimulationS and Analysis of GEne families. This software is a workflow for simulating metagenomic data sets that permits streamlined statistical evaluation of the capabilities of metagenomic methods in variable conditions, including high-novelty communities and short-read sequencing. The workflow is structured as a sequence of Perl modules that enable automated (1) community modeling, (2) simulations of metagenomic sequencing, (3) orientation or translation of reads, and (4) alignment. The second module extends the functionality of MetaSim by adding simple, automated community modeling capabilities, simplifying its command-line use, and adapting it to work better with gene sequences. MetaPASSAGE is the first simulation software to include automated translation of reads and alignment, and it has options to leverage the AMPHORA [14] sequence database and alignment masks by integrating them directly into the workflow. By combining community modeling, metagenomic simulation, and downstream data processing in a single pipeline, MetaPASSAGE enabled our statistical assessment of the accuracy of metagenomic phylogenies in a range of scenarios that accounted for environmental and technological variables, such as phylogenetic algorithm, read length, number of reads in the metagenomic sample, gene family characteristics, and novelty in the metagenomic sample with respect to reference sequences.

### Phylogenetic triangulation enables construction of metagenomic read trees

In order to perform evolutionary analysis of shotgun metagenomic samples without relying on genome or gene assembly, we use an approach to build phylogenetic trees so that each leaf is a short sequencing read from a gene family ("read trees") (see Methods). Our method leverages full-length sequences of the gene (the "reference database") to build a profile model to which reads are aligned. This multiple sequence alignment enables the application of standard phylogenetic methods, which essentially treat reads as examples of full-length gene sequences with a great deal of missing data, as well as placement-type phylogenetic methods. The phylogenetic relationship between two non-overlapping reads is inferred via each of their distances to the full-length sequences. By pruning full-length sequences from the resulting tree, we produce a tree consisting only of reads. The key question is whether the tree is accurate; if read trees reflect the true phylogenetic relationships between shotgun sequences, they open the door to analyses of microbial communities that go beyond enumerating which taxa or genes are present in the sample by relating these individual entities through their evolutionary history.

### Many branch lengths and bifurcations are accurately estimated in metagenomic read trees

To evaluate the accuracy of our approach to constructing read trees, we conducted a simulation study that compares read trees to trees built with the corresponding full-length gene sequences (“source trees”). All simulated datasets are available by request from the authors. We simulated reference databases and samples of metagenomic reads from five gene families, built read and source trees, and quantified phylogenetic errors. We used the normalized Robinson-Foulds (nRF) [19] distance as a measure of topological error and the quantiles of the distribution of branch distortion factors (DF) as a measure of branch-length error (see Methods). The nRF score is very sensitive to mistakes in estimated evolutionary relationships, without regard for branch lengths (two random trees have nRF = 1 with high probability [19]), while the DF distribution reflects over- and under-estimation of branch lengths.

We quantified the impacts of read length (100-bp vs. 400-bp mean), number of reads (50 vs. 200 reads per gene family), reference database size (50 vs. 200 full-length sequences per gene family), reference database diversity (see Methods), gene family characteristics (Table 1), phylogenetic algorithm (RAxML, FastTree, or pplacer), and sequencing error (Illumina error model vs. no sequencing error) on nRF and DF distributions. We studied five gene families: 16S rRNA, the most well characterized microbial gene family, as well as three well-conserved, single-copy protein families covering a range of gene lengths (*rpoB*, *rpsB*, and *dnaG*), and *lolC*, a transmembrane protein family from the ATP-binding cassette (ABC) transporter super family.

**Table 1:** Gene families used in simulations

**Gene name**	**Median length**	**Rate of amino acid evolution**	**Number of sequences**	**Source of sequences**	**Source of profile**
16S rRNA	1535 bp	NA (Highly conserved)	427	RDP	RDP (INFERNAL)
*rpoB*	1296 aa	73.51	460	AMPHORA + GenBank	AMPHORA (HMMER)
*rpsB*	226 aa	51.96	411	AMPHORA + GenBank	AMPHORA (HMMER)
*dnaG*	395 aa	112.53	456	AMPHORA + GenBank	AMPHORA (HMMER)
*lolC*	411 aa	184.04	442	UniProt + GenBank	PhyloFacts (HMMER)

Each family of gene sequences was limited to its unique representatives among AMPHORA taxa (see Methods). Rate of amino acid evolution was determined by summing all branch lengths in a phylogenetic tree inferred via RAxML from the protein sequences; smaller values indicate fewer substitutions and greater conservation. The 16S rRNA gene requires a nucleotide model of evolution and hence has an incomparable value; it is well known to be highly conserved, with variable regions. 16S rRNA sequences were obtained from the Ribosomal Database Project (RDP) [20]. A larger set of 1,071 16S rRNA sequences was used only for the Fast UniFrac analysis (see Additional file 1: Table S1). Amino acid sequences for *rpoB*, *rpsB*, and *dnaG* families were obtained via AMPHORA [14], while corresponding DNA sequences were downloaded from NCBI GenBank [21]. For *lolC*, family members were determined by PhyloFacts [22] (family accession bpg052966 as of February 16, 2011); amino acid sequences were downloaded from UniProt [23], and corresponding DNA sequences were downloaded from EMBL-EBI [24]. Additional file 1: Table S1 provides download dates and sequence accession numbers.

**Figure 2:**
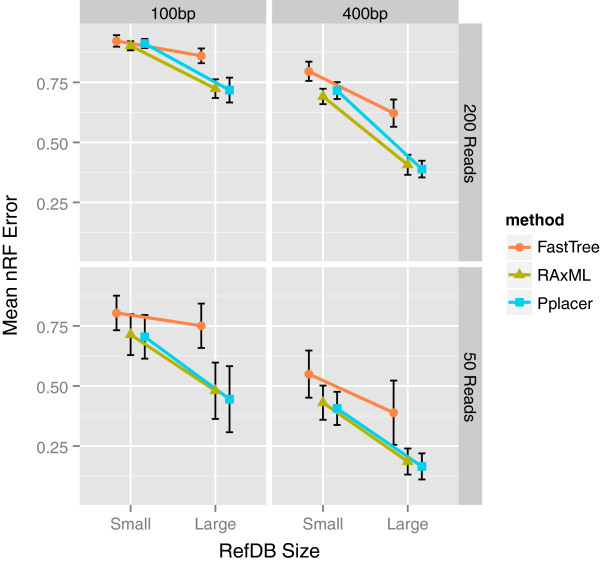
**Read-tree topological error varies with reference database size, read length, and number of reads.** Mean topological error (nRF) varies widely from the most challenging scenario, where the reads are short and there is a relatively large number of reads (upper left), to the inverse scenario (lower right). It is inversely related to both reference database size and read length, and grows with the number of reads. RAxML and pplacer appear to take better advantage of the larger reference database (i.e., the slope of the corresponding line is steeper) than FastTree. In each panel, the nRF measure is averaged over 30 simulations for each combination of simulation parameters. Vertical error bars show a standard deviation above and below the mean. Data for *rpoB* family are shown; trends were similar across gene families tested (Additional file 2: Figure S1).

**Figure 3:**
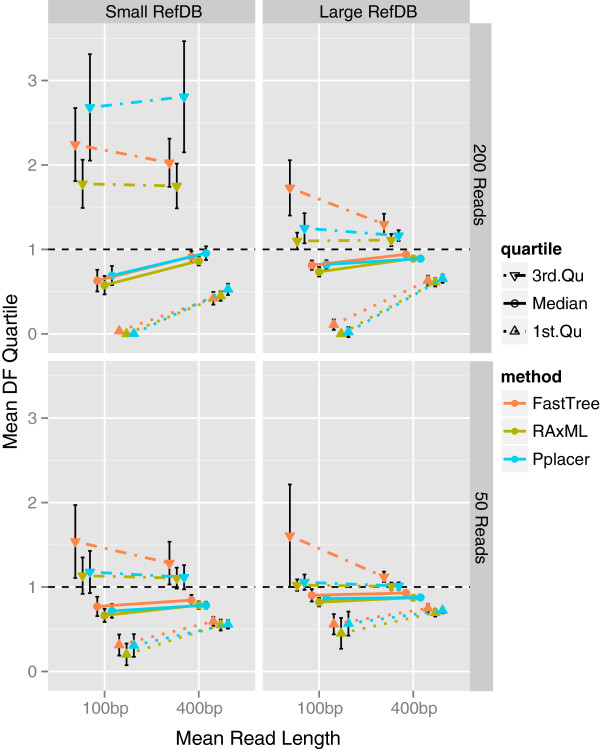
**Branch-length distortion varies with reference database size, read length, and number of reads.** A branch DF of 1.0 is optimal and signifies that a topologically correct branch is estimated to be the same length in the read tree as in the source tree. The amount of branch-length distortion is inversely related to read length and reference database size, and grows with the number of reads. Variation in the DF quartiles indicates that larger reference databases reduce the amount that branch lengths are overestimated (DF third quartile), particularly in scenarios with 200 reads, while longer read lengths consistently improve the amount that branch lengths are underestimated (DF first quartile). In scenarios involving a small reference database, the DF third quartile is increased by increasing the number of reads, regardless of read length; this trend is drastic with pplacer. Each panel shows the mean values of the DF median, first quartile, and third quartile, averaged over 30 simulations for each parameter combination. Vertical error bars show a standard deviation above and below the mean. Data for *rpoB* family are shown; trends were similar across gene families tested (Additional file 3: Figure S2).

As expected, both topological (Figure 2, Additional file 2: Figure S1) and branch-length (Figure 3, Additional file 3: Figure S2) errors tend to be largest in simulations that combine a shorter read length, smaller reference database, and larger number of reads. While minimum and maximum DFs were typically extreme (Table 2), indicating that some branch lengths were very inaccurate, the first and third quartiles of the DF distribution were much closer to 1.0 and more stable across simulations (Figure 3). Quantifying error using normalized branch-score distance (nBS), which accounts for both topological and branch-length errors, showed very similar trends to those seen with the nRF measure (Additional file 4: Figure S3). These results show that for realistic experimental scenarios, bifurcations and branch lengths in shotgun metagenomic read trees can be reasonably estimated.

**Table 2:** **Minimum and maximum DFs for simulations with 200 reads from the *rpoB *gene family**

**Method**	**RefDB size**	**Mean read length**	**Mean min**. **DF**	**Std**. **dev**. **min**. **DF**	**Mean max**. **DF**	**Std**. **dev**. **max**. **DF**
FastTree	Small	100 bp	3.8e-04	1.4e-04	6.9e + 02	8.5e + 02
		400 bp	8.1e-04	4.0e-04	5.3e + 02	3.9e + 02
	Large	100 bp	3.9e-04	1.4e-04	4.6e + 02	3.4e + 02
		400 bp	1.1e-03	7.0e-04	2.0e + 02	1.8e + 02
RAxML	Small	100 bp	2.2e-06	7.8e-07	4.6e + 05	3.4e + 05
		400 bp	5.7e-06	3.3e-06	3.8e + 05	2.3e + 05
	Large	100 bp	2.3e-06	7.2e-07	1.2e + 05	1.5e + 05
		400 bp	7.8e-06	4.2e-06	1.5e + 05	1.9e + 05
Pplacer	Small	100 bp	2.3e-06	5.5e-06	5.4e + 05	4.1e + 05
		400 bp	3.4e-05	3.8e-05	4.7e + 05	2.4e + 05
	Large	100 bp	4.5e-06	7.6e-06	1.5e + 05	1.7e + 05
		400 bp	2.3e-05	4.0e-05	2.0e + 05	2.0e + 05

Minimum and maximum DFs were orders of magnitude away from the ideal value of 1.0, indicating that the most poorly estimated branch lengths in each simulation were typically very inaccurate. Values were somewhat smaller for simulations with 50 reads (data not shown). The mean and standard deviation values of the minimum and maximum DFs are over 30 simulations for each parameter combination. Similar trends were observed for the other gene families.

### Many variables affect read tree accuracy

Nearly every parameter we evaluated in our simulations had an impact on the accuracy of read trees in at least some settings. The most important variables were the reference database, the read length, and the number of reads.

### Effect of reference database

The simulated reference database allowed us to model how read-tree accuracy is influenced by the extent that annotated full-length gene sequences cover the diversity in a gene family. We purposely removed some sequences from the simulated reference database, while still allowing reads to be sampled from these sequences, in order to simulate the commonly encountered situation that a community contains uncharacterized organisms. Comparing simulated reference databases of different sizes and phylogenetic diversity, we found that these variables consistently affect both types of error. The larger reference databases, which contain 43–48% of the sequences in a gene family, result in significantly less topological error (Figure 2, Additional file 2: Figure S1) and branch-length error (Figure 3, Additional file 3: Figure S2) than do smaller reference databases, which contain 11–12% of sequences. Pplacer and RAxML particularly capitalize on the larger reference databases to reduce error. When simulated reference databases of the same size were compared, databases designed to be phylogenetically diverse resulted in slightly less error than random ones (Additional file 5: Figure S4). This relatively minimal effect may be due to the phylogenetic diversity already present in random databases. In practice, existing reference sequences for gene families may be fewer and less diverse than those considered here, so error levels may be higher than those we report. Finally, the trends we observed in the influence of reference database composition on error rates were generally consistent across simulations with different numbers of reads, although the results were most variable in simulations with small numbers of reads (Figure 2, Figure 3, Additional file 2: Figure S1). This finding indicates that it is not merely the proportion of reads relative to reference sequences that determines error rates and patterns.

### Effect of read length

Read length has a pronounced effect on the accuracy of topological (Figure 2, Additional file 2: Figure S1) and branch-length (Figure 3, Additional file 3: Figure S2) inference in all scenarios. Read trees built from 100-bp reads generally have more error and greater variability in error across gene families, regardless of other simulation parameters. In many scenarios, increasing the read length to 400 bp cut the mean topological error by 25–50%. Even when a larger reference database and 200 reads were used, lengthening reads to 400 bp drove mean nRF from as high as 0.7 to below 0.45 for RAxML and pplacer. Branch-length error is also affected by read length; for example, *rpoB* simulations with 200 reads had mean DF first quartiles close to 0 for 100-bp reads, meaning that at least a quarter of topologically correct branches were severely underestimated in length, versus mean DF first quartiles near 0.5 for 400-bp reads (Figure 3). Read length appears to affect the DFs of tip branches much more than that of internal branches (Additional file 6: Figure S5).

### Effect of number of reads

Despite using measures of error that are normalized to adjust for the number of reads in a tree, we observed that topological and branch-length error increased with the number of reads. In some situations, the effect is dramatic; for instance, in the case of a small reference database and 400-bp mean read length, increasing the number of *rpoB* reads from 50 to 200 increased the topological error of all three methods by at least 40% (Figure 2). In scenarios involving a small reference database, the *rpoB* DF third quartile is increased by increasing the number of reads, regardless of read length (Figure 3). This pattern is drastic with pplacer, an effect apparently stemming from relatively high distortion of tip branches (Additional file 6: Figure S5).

### Effect of sequencing error

**Figure 4:**
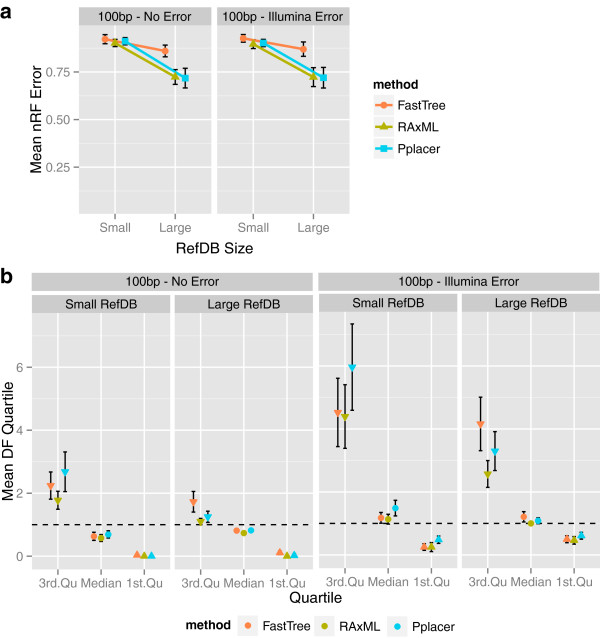
**Comparison of simulations with and without sequencing error.** Sequencing error was simulated using an Illumina-based model. **(a)** Mean topological error (nRF) is not affected by sequencing error. **(b)** Mean values of the DF median, first quartile, and third quartile indicate that sequencing error seems to compensate for the underestimation of branch lengths occurring in simulations without sequencing error. However, some branch lengths are overestimated significantly more in simulations with sequencing error than in those without it. The effect of the reference database is apparent even in the context of sequencing error. In each panel, the error measure is averaged over 30 simulations for each combination of simulation parameters with 200 reads and 100-bp mean read length. Vertical error bars show a standard deviation above and below the mean. Data for the *rpoB* family are shown.

In *rpoB* simulations with 100-bp mean read length and 200 reads, Illumina-like sequencing error has little effect on topological accuracy but does impact branch-length estimation (Figure 4). According to the mean DF first quartile and median, sequencing error may compensate for the underestimation of branch lengths occurring in simulations without sequencing error. However, the mean DF third quartile indicates that some branch lengths are overestimated significantly more in simulations with sequencing error than in those without it. The effect of the reference database is apparent even in the context of sequencing error.

### Effect of phylogenetic algorithm

As has been previously documented, FastTree and pplacer are both capable of analyzing datasets of hundreds of sequences or more within minutes, whereas RAxML is much slower [12,25,26]. At the scale of this study, the difference is not prohibitive for RAxML (depending on available computational resources). With some exceptions, in our study, FastTree’s topological error was typically worse than RAxML’s and pplacer’s, all other factors being equal (Figure 2, Additional file 2: Figure S1); its relatively poor performance is particularly notable in scenarios with short reads or a large reference database. However, in simulations with a smaller reference database and 200 reads, pplacer tends to more severely overestimate branch lengths, i.e., its mean DF third quartiles are much higher than those of the other methods (Figure 3, Additional file 3: Figure S2), regardless of read length. Like pplacer, RAxML is better able than FastTree to leverage the larger reference database to improve topological accuracy, especially with 400-bp reads. One might suspect that RAxML is favored because the fixed reference tree gives it a more restricted optimization landscape. We verified that this is not the case: RAxML performs very similarly when it is not given a fixed reference tree (Additional file 7: Figure S6).

### Effect of gene family

Performance did vary somewhat across the gene families we tested but was not significantly or consistently associated with any variable in Table 1. Overall trends in topological error were generally consistent across gene families (Figure 2, Additional file 2: Figure S1). In scenarios with a larger reference database, the read length had less of an impact on the DF distribution for gene families with shorter sequences (Figure 3, Additional file 3: Figure S2), which we hypothesize is because short reads capture a greater proportion of the variation in the complete sequences.

### Fast UniFrac accurately distinguishes communities using metagenomic phylogenies

**Figure 5:**
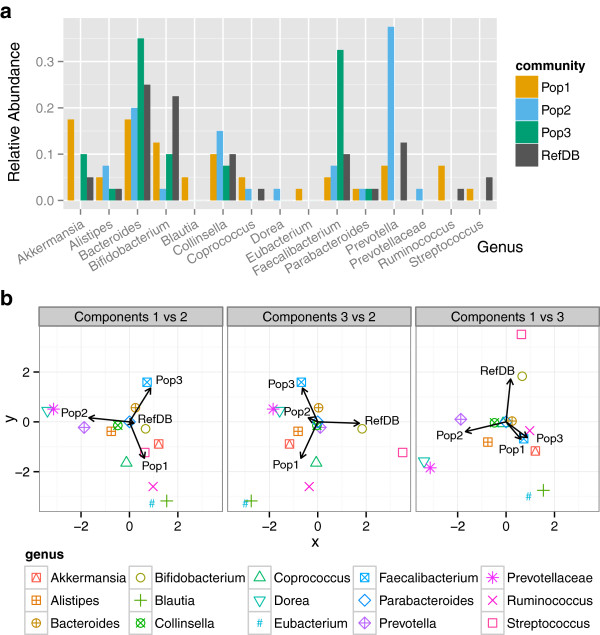
**Simulated communities with distinct relative abundance profiles. (a)** Relative abundances of 15 genera in three simulated communities and the simulated reference database. Pop1 is not strongly dominated by any taxon, but it is has unique taxa (Ruminococcus). Pop2 is dominated by Prevotella, which occurs at much lower levels in the other communities, and lacks Akkermansia. Pop3 is dominated by Bacteroides and Faecalibacterium, which occur at lower levels in the other communities, and lacks Prevotella. **(b)** Correspondence analysis of a matrix giving the number of sequences belonging to each genus in the three simulated communities and reference database (with genera as rows and communities as columns). Each panel shows two of the first three components plotted against each other; these components capture a significant part of the variation among the communities and reference database.

Despite the fact that read trees consistently contain some topological and branch-length errors, we hypothesized that downstream analyses that aggregate information across many branches of a tree might be robust to these errors. To test this hypothesis, we performed additional simulations to mimic metagenomic samples of 16S rRNA sequences from individuals belonging to three types of gut microbiome communities. Depending on other factors, microbiome communities may vary continuously or occupy discrete configurations [3]. Our simulated communities were loosely modeled after those identified previously [27] (see Methods) (Figure 5): one community (“Pop1”) has low levels of a taxon unique to that community (Ruminococcus), while the other two communities are dominated by distinct taxa (Prevotella in “Pop2”, Bacteroides and Faecalibacterium in “Pop3”). We also simulated a reference database that has intermediate levels of most taxa.

**Figure 6:**
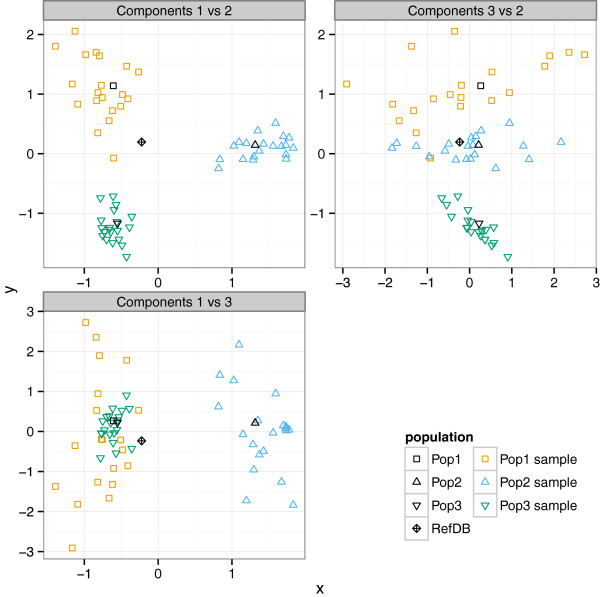
**Simulated metagenomic samples cluster by community; some pairs are much more strongly distinguished than others.** Correspondence analysis of a matrix containing the number of sequences belonging to each genus in simulated shotgun read samples of three simulated communities (with genera as rows and samples as columns), as well as in the simulated communities and simulated reference database. Each panel shows two of the first three components plotted against each other. Although these components capture a large part of the variation among samples and the reference database, read samples of Pop1 and Pop3 are not strongly distinguished by components 1 or 3 (bottom panel), while samples from the other two pairs of communities are easier to distinguish. This analysis is consistent with the performance of weighted Fast UniFrac in distinguishing the three different pairs of communities (see Table 3).

We tested whether an analysis using Fast UniFrac of the read trees from these samples could reliably distinguish the underlying community structure while controlling false positive rates. Since our communities differ critically in relative abundances of taxa, we used the weighted version of the UniFrac metric [28]. First we compared samples from the same community and found that the observed UniFrac false positive rate was consistently at or below the targeted 5% level (Table 3). Next we analyzed samples drawn from all three communities in order to evaluate power. Weighted UniFrac was able to accurately distinguish samples from different community types (Table 3). Variation in performance mainly depended on the types of communities being compared, rather than on the mean read length: Pop1 and Pop3 are much more difficult to distinguish than the other two pairs of communities (Figure 6, Table 3). We did not observe significant differences in false positive rates or power between different phylogenetic methods, despite higher topological and branch-length error with FastTree in many simulations. Thus, we conclude that, while imperfect, metagenomic read trees can be powerful tools for the analysis of microbial communities.

**Table 3:** Accuracy of distinguishing communities using Fast UniFrac on read trees

	**True positive rate** (**sensitivity**)			**False positive rate** (**1**-**specificity**)		
	**FastTree**	**RAxML**	**pplacer**	**FastTree**	**RAxML**	**pplacer**
**Pop 1 vs 2**						
Source Seqs	1.0	1.0	1.0	0.06	0.00	0.03
400-bp Reads	0.8	0.7	0.8	0.06	0.00	0.00
100-bp Reads	0.7	0.7	0.9	0.00	0.00	0.00
**Pop 2 vs 3**						
Source Seqs	1.0	1.0	1.0	0.03	0.00	0.03
400-bp Reads	0.9	0.6	0.9	0.00	0.00	0.00
100-bp Reads	0.8	0.9	0.9	0.06	0.00	0.00
**Pop 1 vs 3**						
Source Seqs	0.0	0.0	0.0	0.03	0.00	0.00
400-bp Reads	0.3	0.0	0.1	0.06	0.00	0.00
100-bp Reads	0.2	0.2	0.2	0.06	0.00	0.00

Weighted Fast UniFrac could reliably distinguish the underlying community structure while controlling false positive rates. The false positive rates, which are based on the null simulations for both communities in each pair, were consistently at or below the targeted 5% level. Weighted UniFrac was able to accurately distinguish samples from Pop1 versus Pop2, and from Pop2 versus Pop3, while it had much less success distinguishing samples from Pop1 and Pop3. Variation in performance was largely independent of mean read length and phylogenetic method.

## Conclusions

We presented and evaluated an approach for conducting evolutionary analyses of shotgun metagenomic data. Our method uses a reference database to guide construction of a metagenomic read tree for a gene family. Using a new simulation pipeline, called MetaPASSAGE, we quantified topological and branch-length errors that occur when building trees of shotgun metagenomic reads versus full-length sequences. Our findings indicate that individual branchings and branch lengths in a metagenomic read tree are reasonably likely to be erroneous, especially if reads are 100 bp or the reference database is not large and diverse. However, many phylogenetic relationships in read trees are accurate, despite how short shotgun reads are compared to full-length gene sequences.

The levels of accuracy we found may not be achievable in cases where the number of reads greatly exceeds that of the reference sequences, e.g., when samples contain a highly abundant or poorly characterized gene family. One potential option, beyond the scope of our study, is to use a hybrid approach that first clusters reads via sequence similarity and then uses representatives from the clusters to build read trees. Less well-characterized gene families, which could not be used in our analysis, may also exhibit different error patterns from those we observed, a possibility consistent with a trend in some of our simulations towards higher error in less conserved genes. Sequencing error may lessen or change read-tree accuracy to some degree, particularly with respect to branch-length estimation, an issue that we study here in the context of Illumina sequencing. The effects of some other features of the data, such as read alignment within hypervariable or conserved regions of a gene family, may be investigated further by extending our modular MetaPASSAGE software.

The results of this study have significant implications for metagenomics research and the development of new methods. When read trees can be reliably built, powerful statistical analyses and comparisons designed for full-length gene sequences (e.g., from eukaryotic genomes) can be directly applied to metagenomics data. Taken in aggregate, our results show that the evolutionary information encoded in a read tree carries almost as much information about community structure, as quantified by weighted Fast UniFrac, as the corresponding source tree. Other approaches that consider an entire tree in order to find large-scale patterns may also be reasonably accurate when applied to metagenomic read trees built with our approach. In contrast, methods that make inferences based on individual branches should be used with caution.

The sheer range of the topological and branch-length error, and its dependence on parameters including read length and reference database diversity, suggest that researchers may be able to influence the applicability of this phylogenetic approach to their projects. Our results provide an initial basis for evaluating trade-offs: for example, the improvement in accuracy obtained from using a larger reference database is in many situations comparable to the improvement obtained from using 400-bp (versus 100-bp) reads. Hence, our study suggests that focusing on gene families with more complete reference databases is especially important when the choice of sequencing technology is limited to shorter reads. Similarly, using the most accurate, but slowest inference method (e.g., RAxML) may be worthwhile for analyzing 100-bp reads or gene families with small reference databases. On the other hand, other inference methods do have competitive accuracy, especially topologically, and are more feasible for large-scale studies. As individual gene families become more deeply characterized, they will be more amenable to analyses based on read trees. Projects such as GEBA [29] that sequence phylogenetically diverse complete genomes are paving the way for more sophisticated methods of analysis.

## Methods

### Building metagenomic read trees

The approach is illustrated in Figure 1. To build a gene family phylogeny in which each leaf is a shotgun read from a metagenomic sample, we first align all reads that have previously been identified as belonging to the family, e.g., with NCBI BLAST (http://blast.ncbi.nlm.nih.gov/) or HMMER (http://hmmer.janelia.org/), to a probabilistic sequence profile for the gene family: a profile hidden Markov Model for proteins or a stochastic context-free grammar for 16S rRNA. These profile models are built from all known full-length sequences for the gene family (“reference sequences”), which are included in the alignment. We apply standard and placement-type phylogenetic algorithms (see below) to the resulting alignment of reference sequences and reads. Finally, we prune the reference sequences from the estimated phylogeny to produce a read tree.

### Direct quantification of error in shotgun read phylogenies

Because metagenomics is a relatively new field, there are no “gold-standard” data sets that would allow us to vary parameters that we expected to influence accuracy. Hence, we designed a large collection of simulated shotgun metagenomic datasets (Figure 1) to assess the effects of read length, phylogenetic method, number of reads, and reference database size and diversity upon the construction of read trees. To create the simulated datasets, we developed and used the MetaPASSAGE software workflow. Details about its implementation are in Additional file 8: Supplementary Methods.

### Selection of genes

To test whether accuracy varies for different gene families, we identified five gene families for which global homology alignments and probabilistic models had already been built and for which at least 400 unique full-length sequences were available (Table 1). We selected 16S rRNA and four proteins: *rpoB*, RNA polymerase beta-subunit encoding gene; *rpsB*, 30S ribosomal protein S2; *dnaG*, a primase for DNA replication; and lipoprotein-releasing system transmembrane protein *lolC*, from the ABC transporter superfamily. This selection enabled us to assess the generality of our findings and identify any impacts of gene length, type (RNA vs. protein), function (housekeeping vs. not), or level of conservation on read-tree construction. For sequence download information and accession numbers, see Table 1 and Additional file 1: Table S1.

### Gene family alignment and quality control

To improve algorithmic performance, we processed the full-length sequences of each gene family to retain only one representative of every subset of identical sequences. For consistency among simulations, we also limited all gene families to the set of taxa represented in AMPHORA. To focus our study on the most common conditions and sources of error, we removed all *Mycoplasma* and *Candidatus* sequences, as these taxa occur in very specific microbiomes and often have extremely fast-evolving sequences, which could present an additional source of inaccuracy. The remaining unique sequences were aligned to the gene-family profile model using INFERNAL [30] for 16S rRNA and HMMER for proteins. For 16S rRNA, we used cmbuild with options “--rf" and “--ere 1.4” to create an INFERNAL profile model from a hand-curated reference alignment obtained from the Ribosomal Database Project [30]. For *rpoB*, *rpsB*, and *dnaG*, we used HMMER2.0 profile models and alignment functionality directly from AMPHORA, which applies a “non-strict” mask designed to improve the alignment quality. For *lolC*, we first downloaded a gene family alignment from PhyloFacts, pruned that alignment appropriately, and, for consistency with the other gene families, created a HMMER2.0 profile model by running hmmbuild with the “-F" option (the “-s” option was also used to create the profile for aligning reads). For all gene families, we removed any empty alignment columns, including those with a single ambiguous character, and any duplicate sequences produced by masking.

### Reference database simulation

To explore the impact of current knowledge of a gene family, we sampled a subset of sequences from the family alignment and generated a new profile model using only these sequences. We call the sampled sequences the simulated reference database. We used two sizes (“small” and “large”, corresponding to 50 and 200 sequences) and, for three of the gene families, two types (“random” and “diverse”) of reference databases. For every combination of size and type, we simulated three reference databases, for a total of 6 (random only) or 12 (random and diverse) databases for each gene family. The random reference databases were constructed by sampling sequences uniformly without replacement. The diverse databases were constructed using available software [29] by maximizing the phylogenetic diversity [31] of half of the sequences and randomly selecting the rest.

### Community simulation

Using the MetaPASSAGE workflow, for each simulation, we composed small or large simulated microbial communities by randomly sampling 50 or 200 full-length sequences from the collection of full-length sequences for each gene family. We call these the source sequences for a given simulation. The number of source sequences corresponded to the sample size in the read simulation step. Source sequences need not be present in the reference database.

### Read simulation

For each gene family, we used MetaPASSAGE to simulate shotgun reads from the sources sequences via the MetaSim software package [17] and automatically process them according to the following protocol. For each combination of mean read length (100 bp and 400 bp), sample size (50 and 200 reads), and random reference database (three “small” and three “large”), we generated ten simulated samples, for a total of 240 datasets. For three gene families, we created 240 additional simulated datasets using the diverse reference databases. No sequencing error was simulated in these datasets in order to accurately quantify the impacts of the other simulated parameters in the absence of experimental measurement error. However, for the *rpoB* gene family, 100-bp mean read length, 200-read sample size, and each of the six random reference databases, we generated ten additional simulated samples, for a total of 60 datasets, using an Illumina-based sequencing error model. As has been done previously [32], this model was extended from the 80-bp Illumina model available on the MetaSim website (http://ab.inf.uni-tuebingen.de/software/metasim/) by repeating the error rate at position 80 an additional 20 bases.

Each simulation began with the community simulation step described above, resulting in a set of source sequences. To produce a realistic distribution of reads across the length of each source sequence, MetaPASSAGE padded both ends of the source sequence with ‘N’s, representing the genome up- and down-stream. It then randomly generated an average of three metagenomic reads (of average length 100 bp or 400 bp) per source sequence and trimmed N’s from the simulated reads. MetaPASSAGE dropped any reads shorter than 50 bp (for the 100-bp mean) or 200 bp (for the 400-bp mean). It oriented (for 16S rRNA) or translated (for protein families) the remaining reads by comparing them with BLAST against the simulated reference database, and removed any reads for which this could not be done accurately. This set of oriented or translated reads was finally filtered so that there remained at most one random read per original source sequence, a step taken to facilitate the direct one-to-one comparison of phylogenies labeled by simulated metagenomic sequences and phylogenies labeled by full-length source sequences. After filtering, MetaPASSAGE aligned the final set of reads with the simulated reference database sequences, using HMMER 2.0 for proteins or INFERNAL 1.0 for RNA sequences. For each simulated dataset, we kept track of which reads came from which source sequences.

### Phylogenetic algorithms

From each alignment of reads to a reference database, we generated read trees using three different phylogenetic inference algorithms: FastTree [25], RAxML [33] (with a fixed reference tree), and pplacer [12]. These represent the range of current approaches in phylogenetics that are computationally feasible for large datasets (related approaches include PhyML [34] and RAxML’s evolutionary placement algorithm [11]; see Additional file 8: Supplementary Methods). We also applied each phylogenetic algorithm to an alignment of the complete set of full-length reference sequences for each gene family. For each simulation, we pruned this tree so that it contained only the leaves labeled by source sequences for the reads in that simulation. We call this tree of source sequences the source tree. Details about the options used with each algorithm are in Additional file 8: Supplementary Methods.

### Performance evaluation

We evaluated each read tree based on how different it was from the corresponding source tree (Figure 1). We compared each pair of trees using normalized versions of standard measures of topological error (normalized Robinson-Foulds distance (nRF)) and branch-length error (normalized branch-score distance (nBS)) [19] (see Additional file 8: Supplementary Methods). The nRF and nBS scores are based on the number of leaf bipartitions occurring in one but not both of the trees. If two trees are identical topologically, nRF = 0. For trees with more than 30 leaves, the expected mean nRF between a pair of random phylogenies is approximately at least 0.99, with a standard deviation of less than 0.0004 [19].

We also developed a new measure, called the distortion factor (DF) distribution, which offers a more refined view of error in branch-length estimation than does nBS for branches that appear in both the read tree and the corresponding source tree, i.e., topologically correct branches. We define the DF of a topologically correct branch as the branch’s length in the read tree divided by its length in the source tree. A branch that is smaller in the read tree than in the source tree has DF < 1.0, meaning its length has been underestimated, and a branch that is larger in the read tree than in the source tree has DF > 1.0, meaning its length has been overestimated. To avoid numerical instability and focus on branches of topological relevance, we computed DFs only for branches with a minimum length (>0.0004) in the source tree, which included 91–96% of branches in the complete gene tree for each gene family. The quartiles of the DF distribution illustrate the extent to which topologically correct branches are typically stretched or shrunk. Formal definitions of all three measures are in Additional file 8: Supplementary Methods.

### Assessment of impact of error in UniFrac-based analyses

We designed a second set of simulations to test the feasibility and accuracy of UniFrac analysis [18] applied to metagenomic read trees. These simulations follow the same general approach as the first set of simulations; unless stated otherwise, the parameter settings are identical.

### Gene family

We used the 16S rRNA gene with the profile model described above and sampled source sequences from a much larger pool of 1,071 full-length reference sequences (Table 1, Additional file 1: Table S1), obtained via fragment recruitment [35] from Human Microbiome Project sequencing of gut samples [36].

### Community types

We simulated three distinct communities (“Pop1”, “Pop2”, and “Pop3”), guided by three “enterotype” communities described by Arumugam et al. [27]. For each community, we defined a relative abundance distribution over 15 genera that fell roughly within the parameters of a corresponding community type in the Arumugam study (Figure 5). In particular, Pop1 does not have an obvious structure but does have low levels of Ruminococcus, a taxon not present in the other communities. Pop2 has a relatively high abundance of Prevotella, while Pop3 has relatively high abundances of Bacteroides and Faecalibacterium. To simulate each community, we then randomly sampled 50 (not necessarly unique) source sequences, distributed over genera according to the community-specific relative abundance distribution, from the set of full-length gene sequences. That set of source sequences was used to generate every sample of simulated reads for that community.

### Reference database simulation

We randomly sampled 33 unique sequences from the same set of full-length gene sequences, using a relative abundance distribution over genera that was intermediate among the simulated populations (Figure 5). This set of reference sequences overlapped with each community and also contained sequences distinct from all three communities.

### Read simulation

Using MetaPASSAGE, we created a total of 60 sets of simulated metagenomic samples: ten sets of simulated metagenomic samples for each combination of community (Pop1, Pop2, and Pop3) and mean read length (100 bp and 400 bp). To produce one sample, MetaPASSAGE first generated 60 simulated metagenomic reads according to the same protocol as described above, except that after orienting the reads, a final random sample of 30 reads was taken, without filtering with respect to source sequences. This number allowed trees to be built and analyzed quickly, while also making it very likely that, for at least some source sequences, multiple reads from the same source would be present (unlike in the previous simulations).

### Phylogenies of combined samples and controls

For a fixed parameter setting (100-bp or 400-bp mean read length), we created a set of alignments that each contained three simulated samples of metagenomic reads and the simulated reference database. To quantify false positives, we generated nine “null test” alignments in which all three samples came from the same community. To quantify true positives, we generated ten alignments in which each of the three samples came from a different community. A read tree was built using each of the three phylogenetic algorithms from each read alignment. Error rates in the analyses of read trees were compared to error rates in the analyses of the corresponding source trees (the “controls”). For each read tree, a control was built with the same phylogenetic method from an alignment containing the unique source sequences.

### Fast UniFrac

We analyzed read trees and source trees using the weighted version of Fast UniFrac. This statistical test is designed to determine whether the samples in a tree come from different communities or similar communities. It assigns a p-value for each of the pairs of samples in each tree. Pairs of samples with low p-values are more likely to be from different communities. For read trees, the “sample id map” used by Fast UniFrac contained a 1 if read *x* appeared in sample *y*, i.e., each read sequence was considered unique. For source trees, the sample id map reported the number of reads simulated from source sequence *x* in sample *y*.

### Performance evaluation

Using a p-value cut-off of 0.05, we analyzed the false positive rate (FPR) and true positive rate (TPR) of Fast UniFrac applied to read trees versus the corresponding source trees (Table 3). The FPR was computed as the proportion of pairs of samples from the null tests incorrectly identified as coming from different communities. The TPR was computed as the proportion of sample pairs from the non-null tests correctly identified as coming from different communities.

## Abbreviations

nRF: Normalized Robinson-Foulds measure of topological tree distance; DF: distortion factor; nBS: Normalized Branch-Score measure of tree distance; TPR: True positive rate; FPR: False positive rate; Min: Minimum; Max: Maximum; Std dev: Standard deviation.

## Competing interests

The authors declare that they have no competing interests.

## Authors’ contributions

SR participated in the study conception and design, programmed the workflow, performed the simulations and analyses, and drafted the manuscript. KP participated in the study conception and design, aided in the analysis and presentation of results, and helped to draft the manuscript. All authors read and approved the final manuscript.

## Supplementary Material

Additional file 1: Table S1 Sequence accession numbers. 16S rRNA sequences were obtained from the RDP on September 01, 2009 as part of a hand-curated alignment; a larger set of 1,071 16S rRNA sequences, used only for the Fast UniFrac analysis, was downloaded from the RDP on November 9, 2010. Amino acid sequences for *rpoB*, *rpsB*, and *dnaG* families were obtained via AMPHORA, and corresponding DNA sequences were downloaded from NCBI GenBank on August 22, 2009 (*rpoB*), May 17, 2011 (*rpsB*), and June 10, 2011 (*dnaG*). For *lolC*, amino acid sequences were downloaded from UniProt on February 16, 2011, and corresponding DNA sequences were downloaded from EMBL-EBI on March 02, 2011. Click here for file

Additional file 2: Figure S1 Trends in topological error are similar across gene families. For all gene families, topological error in read trees is inversely related to both reference database size and read length, and grows with the number or reads. In each panel, the nRF measure is averaged over 30 simulations for each combination of simulation parameters. Vertical error bars show a standard deviation above and below the mean. (Data for *rpoB* family are shown in Figure 2). Click here for file

Additional file 3: Figure S2 DF distributions varied across gene families, but trends were similar. Trends in the variation of DF quartiles with respect to reference database size, mean read length, and phylogenetic method were very similar across gene families, despite differences in their actual values. Each panel shows the mean values of the DF median, first quartile, and third quartile, averaged over 30 simulations for each parameter combination with 200 reads. Vertical error bars show a standard deviation above and below the mean. (Data for *rpoB* family are shown in Figure 3). Click here for file

Additional file 4: Figure S3 Quantifying error using the nBS measure showed similar patterns to those seen with the nRF measure. While the absolute error measured by nBS differed from that of nRF (Figure 2), the patterns across parameter values were very similar. In each panel, the error measure is averaged over 30 simulations for each combination of simulation parameters. Vertical error bars show a standard deviation above and below the mean. Data for *rpoB* family are shown in both panels. Similar trends were observed for other gene families. Click here for file

Additional file 5: Figure S4 Phylogenetic diversity of large reference database is weakly inversely correlated with topological error. The phylogenetic diversity of each reference database was determined by summing all branch lengths in a phylogenetic tree inferred via RAxML from the sequences in that database. Due to their construction (see Methods), our simulated reference databases all have greater diversity than is likely to be present in real reference databases. Each point is the mean of the nRF error over 10 simulations, for 400-bp mean read length and 200 reads. Shadowed region represents the 95% confidence interval. Click here for file

Additional file 6: Figure S5 Tip branch lengths have greater error than internal branches. DF quartiles of tip branches are more extreme than those of internal branches and are affected more by read length, especially in the case of the small reference database. Each panel shows the mean values of the DF median, first quartile, and third quartile, averaged over 30 simulations for each parameter combination with 200 reads, for the *rpoB* family. Vertical error bars show a standard deviation above and below the mean. Click here for file

Additional file 7: Figure S6 RAxML performs similarly regardless of whether it is given a fixed reference tree. Despite the more restricted optimization landscape offered by a fixed reference tree, in our simulations, there was little detectible difference in performance. Here, data for the 16S rRNA gene family are shown. We plot the mean nRF (left) and mean DF quartiles for simulations with 200 reads (right), over 30 simulations for each combination of simulation parameters. Vertical error bars show a standard deviation above and below the mean. Click here for file

Additional file 8: Supplementary Methods Details about software implementation and the methods used in the simulations, phylogenetic inference, error evaluation, and analyses. Click here for file
