# Supplementary figures and images for: Beyond classification: gene-family phylogenies from shotgun metagenomic reads enable accurate community analysis
Source: BMC Genomics. 2013 Jun 22;14:419. doi: 10.1186/1471-2164-14-419 (PMC3701559; doi:10.1186/1471-2164-14-419)

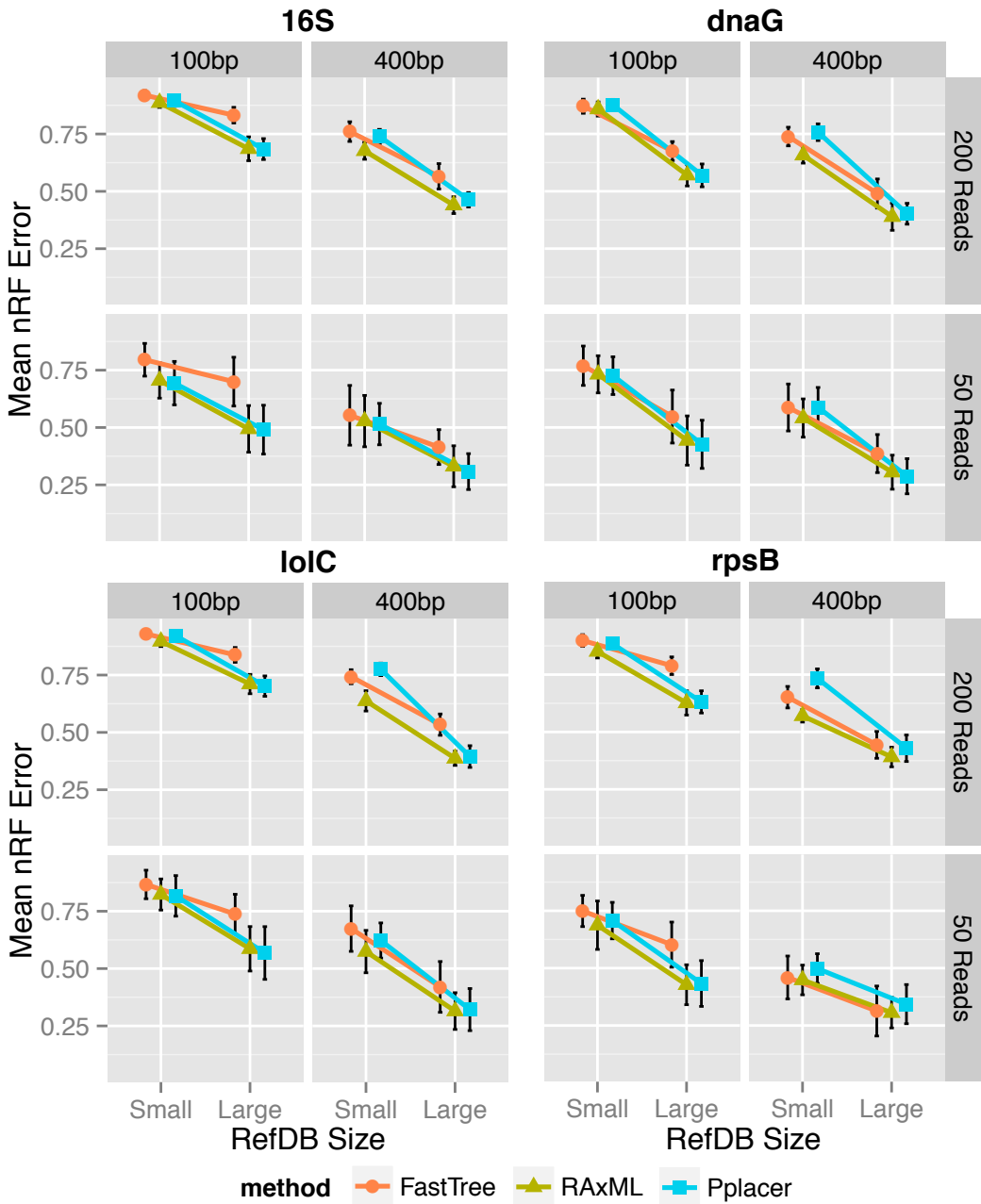

Supplement: Additional file 2: Figure S1 — Trends in topological error are similar across gene families. For all gene families, topological error in read trees is inversely related to both reference database size and read length, and grows with the number or reads. In each panel, the nRF measure is averaged over 30 simulations for each combination of simulation parameters. Vertical error bars show a standard deviation above and below the mean. (Data for rpoB family are shown in Figure 2). [file 1471-2164-14-419-S2.pdf]

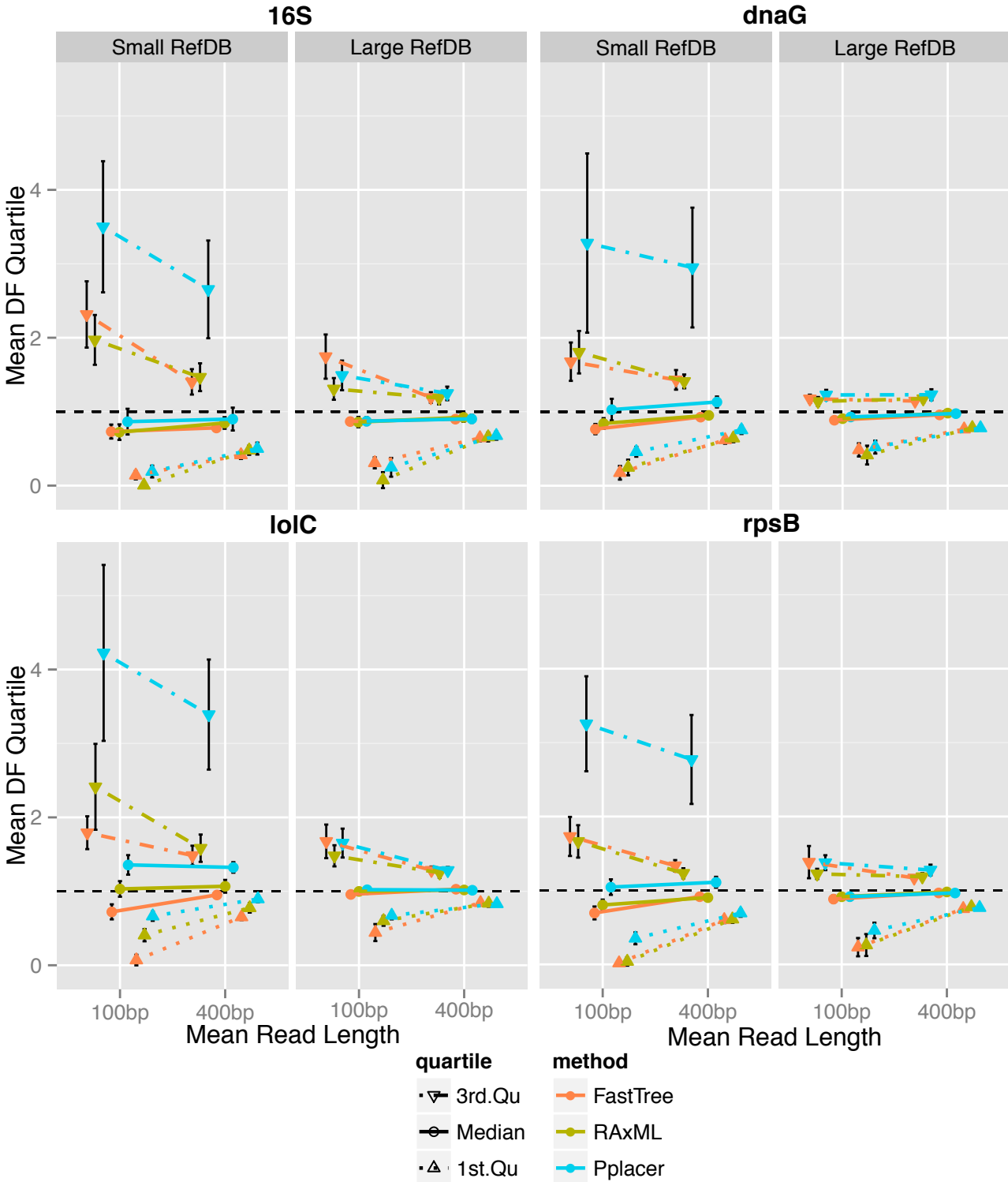

Supplement: Additional file 3: Figure S2 — DF distributions varied across gene families, but trends were similar. Trends in the variation of DF quartiles with respect to reference database size, mean read length, and phylogenetic method were very similar across gene families, despite differences in their actual values. Each panel shows the mean values of the DF median, first quartile, and third quartile, averaged over 30 simulations for each parameter combination with 200 reads. Vertical error bars show a standard deviation above and below the mean. (Data for rpoB family are shown in Figure 3). [file 1471-2164-14-419-S3.pdf]

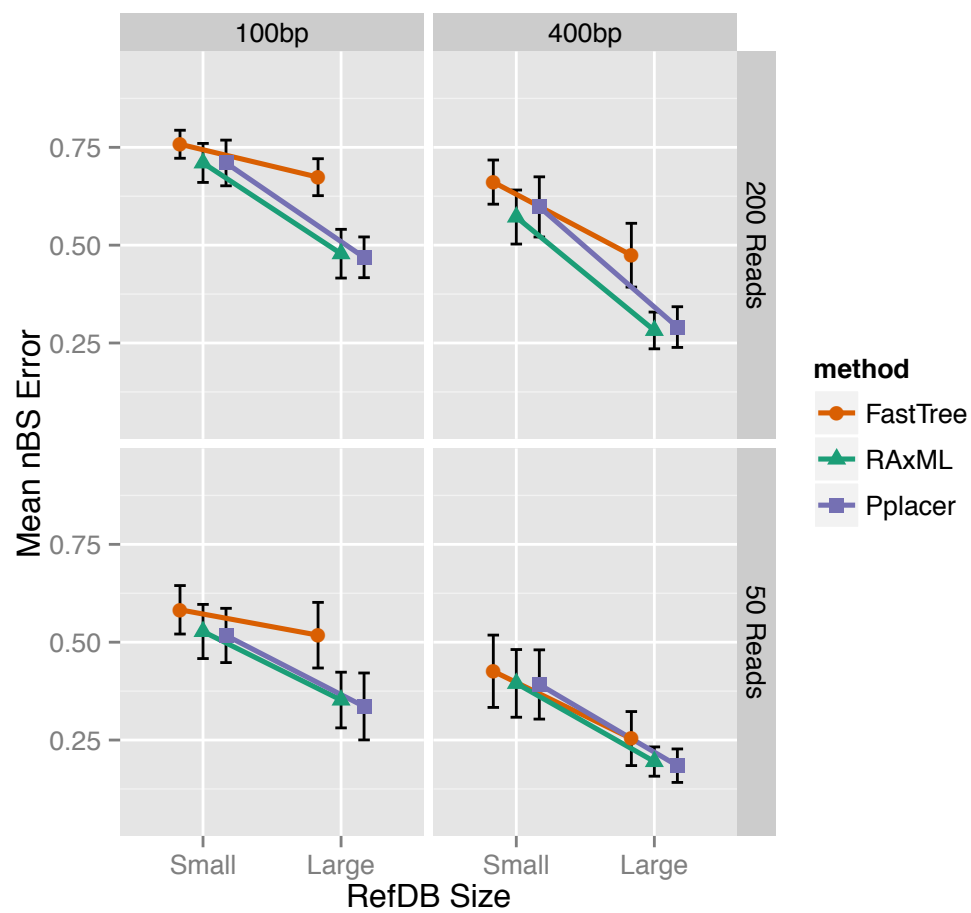

Supplement: Additional file 4: Figure S3 — Quantifying error using the nBS measure showed similar patterns to those seen with the nRF measure. While the absolute error measured by nBS differed from that of nRF (Figure 2), the patterns across parameter values were very similar. In each panel, the error measure is averaged over 30 simulations for each combination of simulation parameters. Vertical error bars show a standard deviation above and below the mean. Data for rpoB family are shown in both panels. Similar trends were observed for other gene families. [file 1471-2164-14-419-S4.pdf]

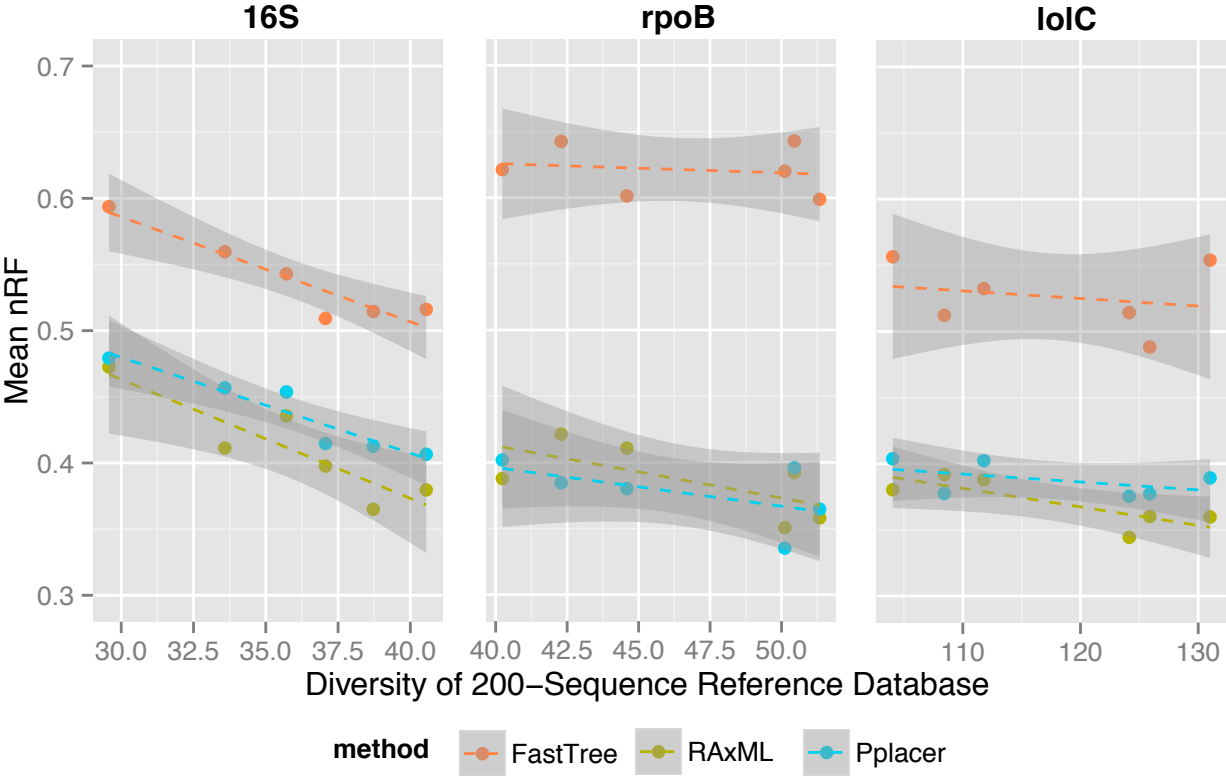

Supplement: Additional file 5: Figure S4 — Phylogenetic diversity of large reference database is weakly inversely correlated with topological error. The phylogenetic diversity of each reference database was determined by summing all branch lengths in a phylogenetic tree inferred via RAxML from the sequences in that database. Due to their construction (see Methods), our simulated reference databases all have greater diversity than is likely to be present in real reference databases. Each point is the mean of the nRF error over 10 simulations, for 400-bp mean read length and 200 reads. Shadowed region represents the 95% confidence interval. [file 1471-2164-14-419-S5.pdf]

# Tip Branches

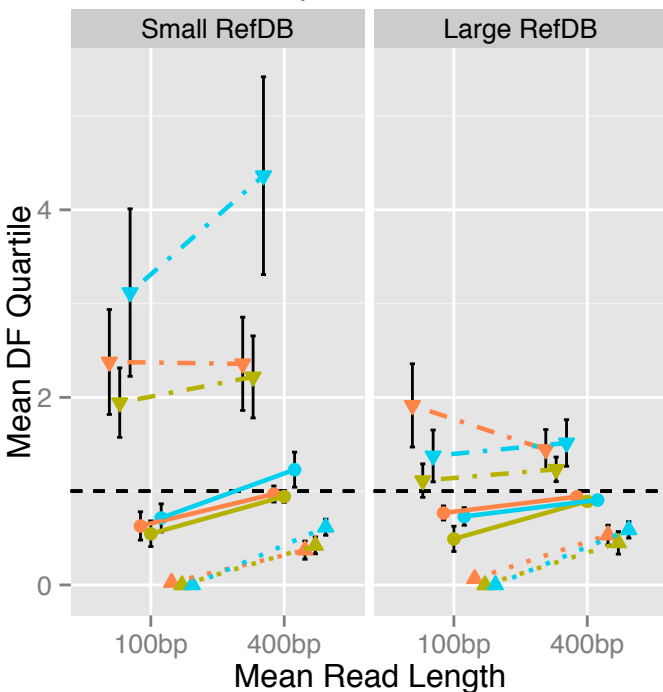

# Internal Branches

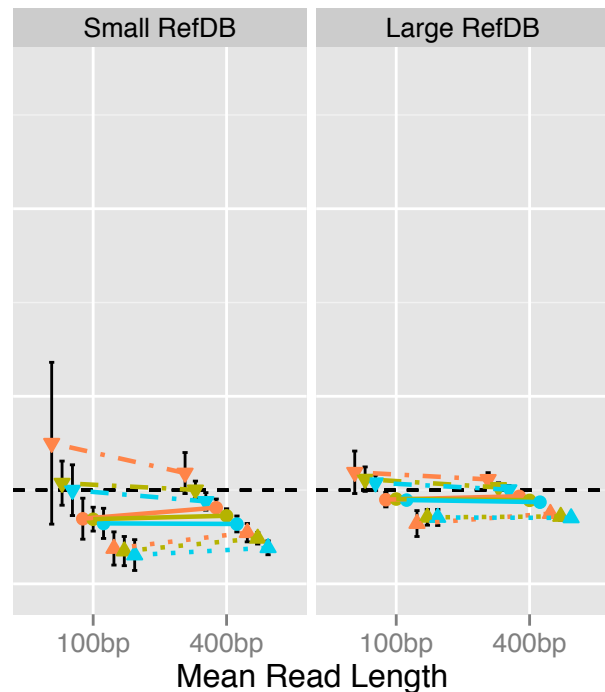

Supplement: Additional file 6: Figure S5 — Tip branch lengths have greater error than internal branches. DF quartiles of tip branches are more extreme than those of internal branches and are affected more by read length, especially in the case of the small reference database. Each panel shows the mean values of the DF median, first quartile, and third quartile, averaged over 30 simulations for each parameter combination with 200 reads, for the rpoB family. Vertical error bars show a standard deviation above and below the mean. [file 1471-2164-14-419-S6.pdf]

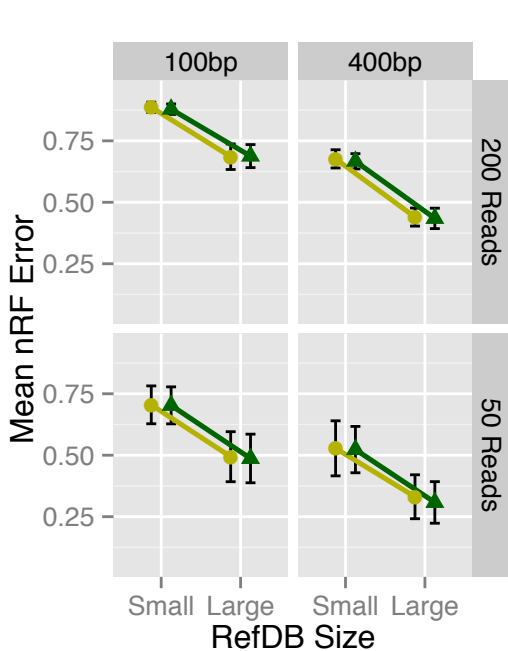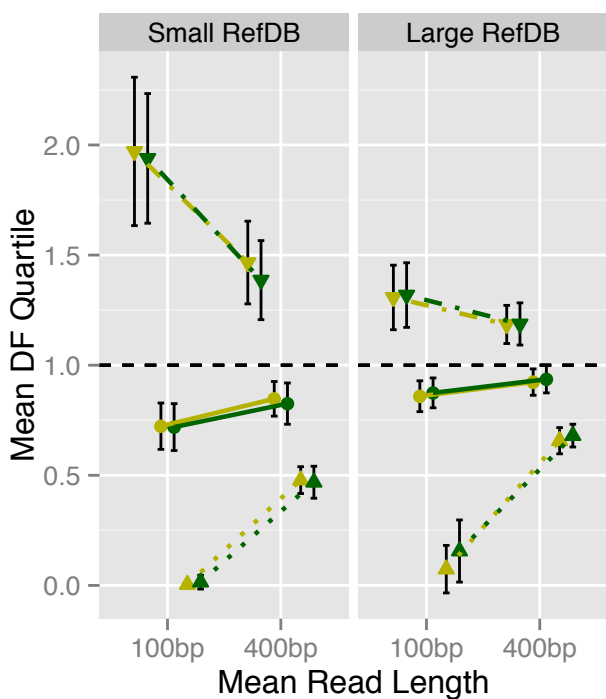

Supplement: Additional file 7: Figure S6 — RAxML performs similarly regardless of whether it is given a fixed reference tree. Despite the more restricted optimization landscape offered by a fixed reference tree, in our simulations, there was little detectible difference in performance. Here, data for the 16S rRNA gene family are shown. We plot the mean nRF (left) and mean DF quartiles for simulations with 200 reads (right), over 30 simulations for each combination of simulation parameters. Vertical error bars show a standard deviation above and below the mean. [file 1471-2164-14-419-S7.pdf]
