# Supplementary material for: Beyond classification: gene-family phylogenies from shotgun metagenomic reads enable accurate community analysis
Source: BMC Genomics. 2013 Jun 22;14:419. doi: 10.1186/1471-2164-14-419 (PMC3701559; doi:10.1186/1471-2164-14-419)
Supplement: Additional file 8: Supplementary Methods — Details about software implementation and the methods used in the simulations, phylogenetic inference, error evaluation, and analyses. [file 1471-2164-14-419-S8.doc]

## Supplemental Methods

### Phylogenetic Algorithms

We investigated read trees built with three phylogenetic algorithms.

FastTree uses a combination of minimum-evolution and maximum-likelihood heuristics with an approach based on neighbor joining to infer phylogenies efficiently on large sets of sequences. FastTree, version 2.1.3, was run with the “-pseudo” flag on the alignments containing simulated reads and without the “-pseudo” flag on the reference alignments.

One of two leading maximum-likelihood methods (the other is PhyML), RAxML is widely recognized as achieving an excellent trade-off in accuracy versus efficiency, though it is significantly slower than NJ methods. RAxML, version 7.2.6, was used with the PROTCATJTT model for proteins and the GTRCAT model for 16S rRNA. The “-r” option constrained tree topology with a fixed reference tree, which was built using the same model from an alignment of the simulated reference database.

Pplacer was chosen as a representative of two new evolutionary placement methods (the other is RAxML’s evolutionary placement algorithm), which classify metagenomic reads by optimizing the independent placement of each read on a fixed phylogenetic reference tree. Pplacer, version v1.1.alpha08 was run with the “-t” and “-s” options to specific the reference tree file and RAxML “info” file, respectively. The pplacer reference tree was built using RAxML, version 7.2.6, using the PROTGAMMAJTT model for proteins and the GTRGAMMA model for 16SrRNA, from an alignment of the simulated reference database. The placement files created by Pplacer were converted to trees using the “guppy tog” program in the pplacer software suite.

Error measures

We used the following measures to quantify differences between read and source trees.

**Normalized Robinson-Foulds Distance (nRF):** Every branch in a tree defines a bipartition of the leaves via removal of the branch. Suppose all possible m leaf bipartitions are enumerated. Then a tree *X* can be uniquely represented by a vector *I^X^*=(*I*_1_*^X^*,*I*_2_*^X^*,…,*I_m_^X^*) such that *I_i_^X^* is 1 if bipartition *i* corresponds to a branch in tree *X*, and 0 otherwise. For a read tree *X* and corresponding source tree *Y*, each with *n* leaves, we define the topological error of *X* based on the widely used Robinson-Foulds measure of topological distance between *X* and *Y*, i.e., the number of bipartitions occurring in exactly one of the trees. Since non-normalized measures of phylogenetic distance typically increase with the number of leaves in the tree, we used a normalized version, the nRF measure, based on Steel and Penny, which divides by the maximum possible value (for *n* leaves):

.

Values of nRF tend to increase from zero rapidly with any topological differences.

**Normalized Branch-Score Distance (nBS):** The standard branch-score distance is a measure of both topological and branch-length distance that is related to the Robinson-Foulds distance. Each tree is represented by a vector *X*=(*x*_1_, *x*_2_, …, *x_m_*) such that *x_i_* is the length of the branch corresponding to bipartition *i* (0 if that branch does not occur in the tree). The branch-score distance is the square root of the sum of the squares of the entries in the difference between the two vectors. Our normalized version, based on a proposal by Steel and Penny, measures the error of a read tree *X* with respect to a source tree *Y* by dividing their branch-score distance by the maximum possible distance between two non-negative vectors:

.

**Distortion Factor (DF) Distribution:** To get a finer-grained view of the error in branch-length estimation, we defined the distortion factor (DF) of a tree branch that appears in both the read tree *X* and the corresponding source tree *Y* as the branch’s length in *X* divided by its length in *Y*. That is, for a leaf bipartition *i* such that *x_i_*≠0 and *y_i_*≠0,

$DF(i)=\frac{x_{i}}{y_{i}}$.

We then consider the distribution of the distortion factor over all branches that appear in both the read tree and the source tree, i.e., over all branches that are considered to be correct and do not contribute to the nRF error.

### Implementation of MetaPASSAGE Workflow

Implemented as a set of Perl program modules and scripts, MetaPASSAGE is designed to be a flexible and modular approach to larger-scale metagenomic simulations with automated read processing. The extensive command-line options that arise from this flexibility are simplified via default settings. Comprehensive documentation and examples are provided (https://github.com/sriesenfeld/MetaPASSAGE). Advanced users can modify the program to adapt its functionality.

**Input Data.** The main input at the start of the workflow is a FASTA file of full-length gene or genome sequences, which we call the reference sequences. Protein sequences may be input as two separate files of corresponding amino acid and DNA sequences, and RNA sequences are input as a single file of DNA sequences. (Utility scripts included translate RNA to DNA and back.) Alternatively, if the workflow is entered at a later stage, the inputs required for only that stage may be provided. MetaPASSAGE also has a specialized interface with the AMPHORA software package, which includes sequences and curated alignments for a group of protein families.

**Simulated Reference Database.** To help evaluate the downstream effects of available reference sequences on an analysis, MetaPASSAGE can make use of a simulated reference database, which is a subset of the full-length reference sequences, in community modeling and processing of simulated reads. To randomly generate a simulated reference database or to create one that contains a phylogenetically diverse subset of sequences, MetaPASSAGE includes a specialized script that samples from the input reference sequences, guided, optionally, by a phylogenetic tree. This same script can also be used to automate retrieval of sequences from the NCBI RefSeq database; it eases, in particular, retrieval of the DNA sequences corresponding to protein sequences in the AMPHORA database.

**Community Models**. By default, to simulate the sequences present in an individual community, MetaPASSAGE takes a uniform random sample of a user-specified number of sequences from the input reference sequences. Command-line options can modify this process to simulate communities containing few abundant species and many rare ones, or to constrain the sampling in various ways. The desired amount of overlap between the community and the simulated reference database can be specified, or limits can be set for the number of sequences sampled from specific genera or any other category used to label the input sequences. The output is a taxonomic profile of the type used by MetaSim to specify the relative abundance of each sequence in the simulated community (which can also be created and input independently), and a FASTA file of the distinct, full-length, DNA sequences.

**Running MetaSim**. The essential functionality of MetaSim is to produce a set of simulated metagenomic reads from an input set of distinct full-length DNA sequences and a taxonomic profile, according to a given sequencing model, such as Sanger or 454 shotgun sequencing, or their own “empirical” sequencing model. To facilitate running the command-line version of MetaSim, MetaPASSAGE contains a wrapper module that calls MetaSim on the output of the community modeling step. This wrapper controls how MetaSim is run by setting defaults for several parameters, including sequencing options (easily changed), so as to avoid Metasim hanging or halting with errors, which can be caused by the interaction of parameter settings. As a basic measure of quality control, a read-length threshold can be specified in MetaPASSAGE, in which case simulated reads that are shorter than the specified threshold are automatically discarded. The output of this stage is a FASTA file of simulated shotgun metagenomic DNA sequence reads.

To improve the functionality of MetaSim with gene families, the MetaPASSAGE wrapper includes an option for padding gene sequences on each end with ‘N’s before they are passed to command-line MetaSim, which assumes that input sequences are circular genomes. The padding ensures that simulated reads are distributed across the ends of the gene sequence, approximately as they would be in whole-genome shotgun sequencing. MetaPASSAGE automatically removes padding after simulated sequencing, and it checks for and discards malformed reads that bridge a sequence of ‘N’s.

**Read Orientation/Translation**. To prepare simulated DNA reads for downstream analysis, a MetaPASSAGE module automatically orients or translates them. Using NCBI BLAST (http://blast.ncbi.nlm.nih.gov/), reads are compared against a BLAST database, which is formatted automatically from the simulated reference database or, if unspecified, the full set of reference sequences. (An independently created BLAST database can also be specified.) MetaPASSAGE uses the BLAST expect values of the hits to determine the best orientation or frame of translation for each read. As in real sequencing experiments, reads from “novel” sequences in a community, i.e., sequences with poor representation in the simulated reference database, may not get oriented or translated correctly. If both amino acid and corresponding DNA sequence files are given as the simulated reference database, the BLAST database is created from the amino acid version, and the blastx program is used; otherwise, blastn is used. In the former case, reads are then translated into amino acid sequences using EMBOSS transeq (http://emboss.sourceforge.net/apps/). If no hit is clearly best, the read may be oriented in both directions or translated in multiple frames. Reads without a hit scoring above a certain threshold are not included in the output. The BLAST output may be optionally saved, and BLAST parameters may be optionally set by the user. To reduce the number of reads, the user may provide a target number of reads to be randomly sampled from the set of fully processed reads that have passed quality control measures. Or the output may be filtered so that there is at most one read for each original full-length sequence, which simplifies a direct comparison of results obtained analyzing full-length sequences versus reads. The main output is a FASTA file of oriented DNA reads or translated amino acid reads.

**Alignment.** To facilitate the testing of inference methods designed for individual gene families, the final stage of MetaPASSAGE produces an alignment of the oriented or translated simulated reads, optionally including the simulated reference database. The main input required, in addition to the reads, is a probabilistic profile model, i.e., either a hidden Markov model (HMM) of the type used by HMMER (<http://hmmer.janelia.org/>) for protein sequences or a covariance model of the type used by INFERNAL for RNA sequences. HMMER or INFERNAL are used to align the reads and, optionally, the simulated reference database to the profile model. The output alignment format depends on the alignment method; utility scripts are provided for translating between formats.
